# Convergent Evolution in Breadth of Two V_H_6-1-Encoded Influenza Antibody Clonotypes from a Single Donor

**DOI:** 10.1016/j.chom.2020.06.003

**Published:** 2020-09-09

**Authors:** Nicholas C. Wu, Sarah F. Andrews, Julie E. Raab, Sarah O’Connell, Chaim A. Schramm, Xintao Ding, Michael J. Chambers, Kwanyee Leung, Lingshu Wang, Yi Zhang, John R. Mascola, Daniel C. Douek, Julie E. Ledgerwood, Adrian B. McDermott, Ian A. Wilson

**Affiliations:** 1 Department of Integrative Structural and Computational Biology, The Scripps Research Institute, San Diego, CA 92037, USA; 2 Vaccine Research Center, National Institute of Allergy and Infectious Diseases, National Institutes of Health, Bethesda, MD 20892, USA; 3 The Skaggs Institute for Chemical Biology, The Scripps Research Institute, San Diego, CA 92037, USA

**Keywords:** influenza, hemagglutinin, antibody, stem, vaccine, crystal structure, somatic hypermutation

## Abstract

Understanding how broadly neutralizing antibodies (bnAbs) to influenza hemagglutinin (HA) naturally develop in humans is critical to the design of universal influenza vaccines. Several classes of bnAbs directed to the conserved HA stem were found in multiple individuals, including one encoded by heavy-chain variable domain V_H_6-1. We describe two genetically similar V_H_6-1 bnAb clonotypes from the same individual that exhibit different developmental paths toward broad neutralization activity. One clonotype evolved from a germline precursor recognizing influenza group 1 subtypes to gain breadth to group 2 subtypes. The other clonotype recognized group 2 subtypes and developed binding to group 1 subtypes through somatic hypermutation. Crystal structures reveal that the specificity differences are primarily mediated by complementarity-determining region H3 (CDR H3). Thus, while V_H_6-1 provides a framework for development of HA stem-directed bnAbs, sequence differences in CDR H3 junctional regions during VDJ recombination can alter reactivity and evolutionary pathways toward increased breadth.

## Highlights

• Two V_H_6-1 influenza HA stem-specific clonotypes are isolated from one individual
• Precursors for these two V_H_6-1 clonotypes bind different influenza A HA groups
• Differences in the CDRH3 conformation mediate the distinctive binding profiles
• Somatic hypermutation leads to similar binding breadth between the two clonotypes

## Graphical Abstract

**Figure:**
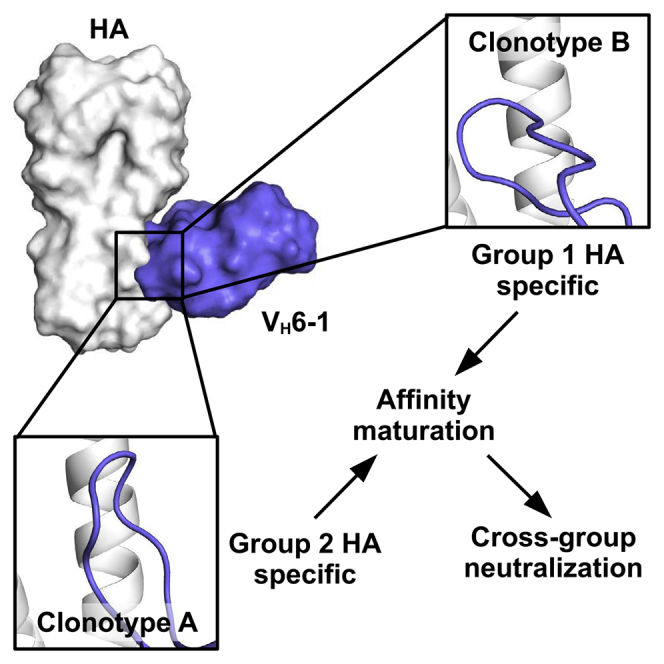

## Teaser

Wu et al. describes two genetically similar influenza antibody clonotypes from the same individual. Clonotype precursors recognize different influenza A HA groups, but both develop cross-group neutralizing capability through affinity maturation. Structural characterization reveals that the specificity difference between clonotypes is mainly due to conformational variation in CDR H3.

## Introduction

Despite being studied extensively for over 90 years, influenza virus remains a global health concern. Seasonal influenza vaccination is the only preventive measure currently available but often has limited efficacy (Belongia et al., 2016). Since the antibody responses elicited by seasonal influenza vaccination are usually strain-specific, the vaccine has to be updated annually to keep pace with the evolution of circulating strains. Furthermore, the seasonal vaccine offers little protection against potential influenza pandemics. The ultimate solution is a universal influenza vaccine that offers broad protection across the enormous diversity of influenza strains, subtypes, and types (influenza A and B) (Jang and Seong, 2019; Morens and Taubenberger, 2019; Nachbagauer and Palese, 2020; Paules et al., 2017). Over the past decade, many broadly neutralizing antibodies (bnAbs) have been identified and characterized that target the influenza hemagglutinin (HA) glycoprotein, the major surface antigen of influenza virus (Wu and Wilson, 2017). Discovery of such bnAbs in humans after natural infection or vaccination has convincingly demonstrated that broad antibody responses can indeed be elicited against influenza virus (Wu and Wilson, 2019) and revitalized the possibility of pursuing a universal influenza vaccine (Corbett et al., 2019; Impagliazzo et al., 2015; Krammer et al., 2013; Yassine et al., 2015).

A total of 16 subtypes of influenza HA exist in the avian reservoir and can be classified into antigenic group 1 (H1, H2, H5, H6, H8, H9, H11, H12, H13, and H16) and group 2 (H3, H4, H7, H10, H14, and H15). HA is a trimeric glycoprotein that is critical for viral entry and is composed of a globular head domain atop a more elongated stem domain. The HA head domain contains the receptor-binding site that engages sialoside receptors on host cells and is highly diverse among subtypes, whereas the stem domain carries the machinery for virus-host membrane fusion and is relatively conserved. For this reason, antibodies that target the HA stem usually have a higher breadth than those against the HA head, even those that specifically target the receptor-binding site (Zost et al., 2019).

The central premise of a universal influenza vaccine is to elicit potent and broadly neutralizing and potent Abs that can protect against seasonal, emerging, and pandemic viruses and, therefore, requires a fundamental understanding of how such bnAbs develop from naive unmutated precursors. Human cross-group HA stem-directed bnAbs can be elicited from natural infection or from different vaccination regimens, such as the seasonal influenza vaccine (Corti et al., 2011; Dreyfus et al., 2012; McCarthy et al., 2018; Nakamura et al., 2013), H5N1 vaccine (Andrews et al., 2017; Joyce et al., 2016), and H7N9 vaccine (Andrews et al., 2017). These bnAbs often share genetic elements across individuals (Andrews and McDermott, 2018), likely because the formation of cross-group HA stem-directed bnAbs is molecularly challenging due to group- and subtype-specific structural features in the HA stem (Zost et al., 2019). There are several examples of HA stem-directed bnAbs found in multiple people that are composed of similar genetic elements and have specific amino-acid motifs critical for binding referred to as convergent immunoglobulin classes (Andrews and McDermott, 2018; Joyce et al., 2016). Each of these classes is thought to evolve from a germline precursor with limited breadth that gains broad reactivity across HA groups upon somatic hypermutation. However, only a limited number of studies have examined how cross-group bnAbs evolve from germline precursors (Fu et al., 2016; Kallewaard et al., 2016; McCarthy et al., 2018). Thus, we know little about the development of influenza bnAbs in humans.

Here, we investigate the development and mode of binding of two independent cross-group HA stem-directed bnAb clonotypes (A and B) arising from different naive precursor cells, but isolated from the same individual who received both H5N1 and H7N9 vaccines. Both clonotypes were encoded by the same V_H_, D_H_, and V_κ_ germline genes, with the same complementarity-determining region (CDR) H3 and CDR L3 lengths but with unique V_H_D_H_J_H_ and V_K_J_K_ junctions. Despite the similarity in germline usage, the inferred early intermediates of these two clonotypes exhibited different preferences for binding HA subtypes from the two influenza A groups. However, both clonotypes evolved to acquire cross-group reactivity. Crystal structures of these antibodies in complex with influenza HA revealed that the conformation of CDR H3 is a major determinant of this HA specificity. Our analysis further delineates how the acquired somatic mutations lead to affinity maturation and evolution of breadth. In summary, this study provides important insight into the evolutionary pathways of bnAbs and suggests that immunogens representing both influenza A group 1 and group 2 HA can efficiently drive increased breadth of the B cell response.

## Results

### Two V_H_6-1/V_κ_3–20-Encoded Antibody Clonotypes from a Single Donor

**Figure 1:**
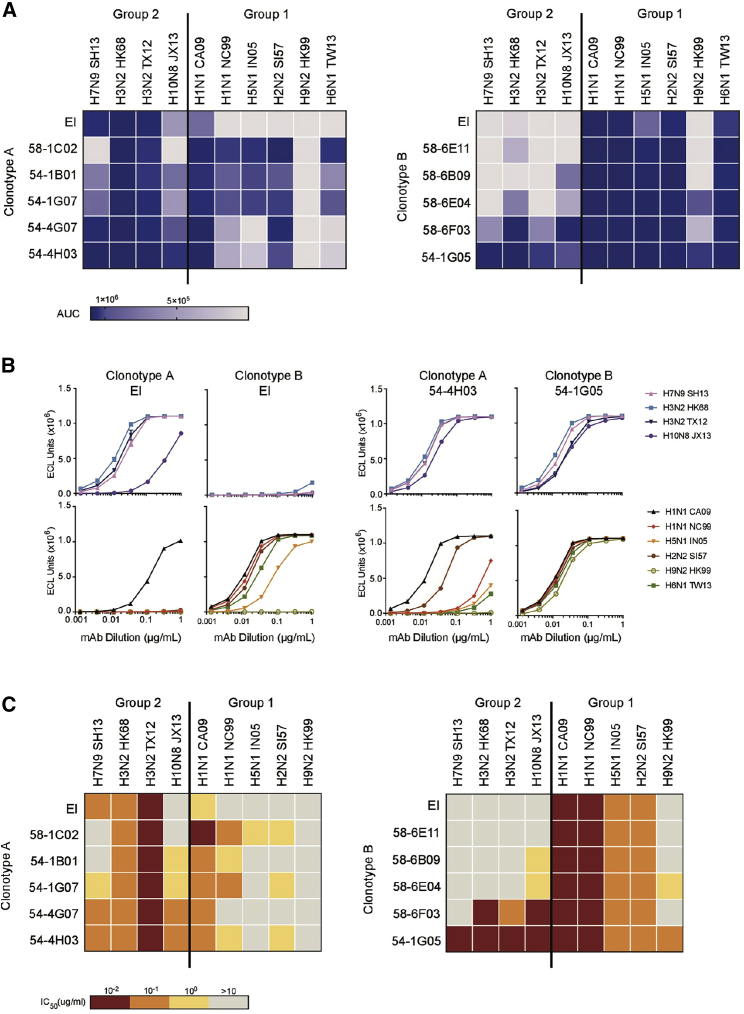
Reactivity Profile of Two V_H_6-1/Vκ3-20-Encoded Antibody Clonotypes from a Single Donor (A and B) mAbs were tested for binding by meso scale discovery (MSD) to recombinant HA from 10 influenza strains as indicated. Full influenza strain names are detailed in STAR Methods. (A) The area under the binding curve (AUC) of each mAb for each HA is indicated by different colors as shown in the legend. (B) Binding curves of representative mAbs are shown. EI, early intermediate. Data are representative of two independent experiments. (C) The mAbs were tested for the ability to neutralize the indicated influenza strains in a pseudovirus assay. mAbs are color-coded according to the neutralization IC_50_ as indicated in the legend. Data are representative of two independent experiments.

Two HA stem-directed bnAb clonotypes, A and B, were elicited upon vaccination with H5N1 and H7N9 from a single donor (Andrews et al., 2017). Both clonotypes were encoded by germline genes V_H_6-1, D_H_3-3, and V_κ_3-20 with the same CDR H3 and CDR L3 lengths (Figures S1A and S1B). However, despite these genetic similarities, they had unique binding and neutralization profiles across HA subtypes. Members of clonotype A preferentially bound group 2 HA, whereas members of clonotype B primarily recognized group 1 HA (Figure 1A; Table S1). Indeed, clonotype A was primarily elicited by vaccination with group 2 H7N9, while clonotype B dominated upon vaccination with group 1 H5N1 (Figure S1C) (Andrews et al., 2017). Next, we synthesized and expressed an early intermediate for each clonotype. The sequences of early intermediates were composed of the germline V gene and the inferred most recent common ancestor CDR3 based on a maximum-likelihood phylogenetic tree constructed from heavy and light chain sequences. In agreement with the binding profiles of isolated clones from each clonotype, the early intermediate of clonotype A primarily recognized group 2 HA, whereas the early intermediate of clonotype B was specific to group 1 HA (Figures 1A and 1B). When we investigated the ability of these clonotypes to neutralize influenza subtypes in a pseudovirus assay, a similar pattern was observed. While members of clonotype A primarily neutralized group 2 subtypes, clonotype B members primarily neutralized group 1 subtypes (Figure 1C). Nonetheless, both clonotypes gave rise to bnAbs that could bind and neutralize both group 1 and 2 HAs. For example, 54-4H03 from clonotype A had not only strong neutralization activity to group 2 (H3, H7, and H10) subtypes, but also strong to moderate neutralization to group 1 HAs (H1 CA09 and H2) (Figures 1B and 1C). Similarly, 54-1G05 from clonotype B was able to bind both group 1 (H1, H2, H5, H6, and H9) and group 2 (H3, H7, and H10) subtypes and exhibited high neutralization titers across both groups (Figures 1A–1C). Thus, we observe convergent evolution in the breadth of two clonotypes originating from germline ancestors with distinct HA specificities. Of note, some members in clonotype A (e.g., 54-1B01 and 54-4G07) showed reasonable cross-binding activity to H2 and/or H5 HAs without detectable neutralizing activity. Such a seemingly discordant result has been described for other HA stem-directed bnAbs (Andrews et al., 2017; Dreyfus et al., 2012). Nevertheless, the underlying mechanism will require further investigation.

### Crystal Structures of 54-4H03 and 54-1G05

**Figure 2:**
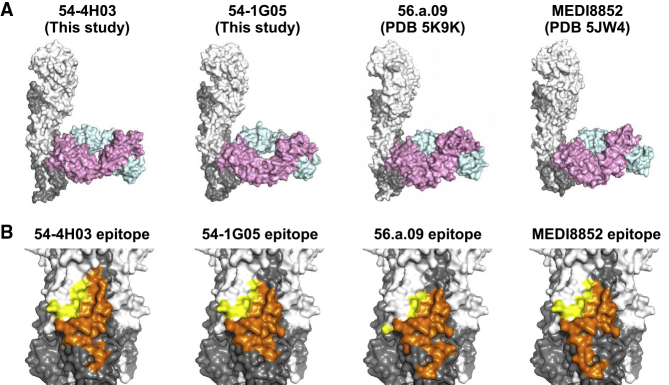
Crystal Structures and Epitopes of 54-1G05 and 54-4H03 (A) Comparison of the binding of Fabs 54-1G05 (this study), 54-4H03 (this study), 56.a.09 (PDB 5K9K) (Joyce et al., 2016), and MEDI8852 (PDB 5JW4) (Kallewaard et al., 2016) to HA in crystal structures. HA1 is colored in white, HA2 in gray, Fab heavy chain in pink, and light chain in cyan. Only one protomer of the HA trimer is shown. (B) Comparison of the epitopes of 54-1G05, 54-4H03, 56.a.09, and MEDI8852. Yellow, epitope residues on HA1; orange, epitope residues on HA2.

Previous studies have described several HA stem-directed bnAbs that are encoded by V_H_6-1 (Joyce et al., 2016; Kallewaard et al., 2016). However, these V_H_6-1-encoded bnAbs all arose from a group 1-specific germline ancestor, making clonotype A unique among these HA stem-directed antibodies. To better understand the binding mechanism of clonotype A compared with that of clonotypes with a group 1 origin, we performed X-ray crystallography analysis. Crystal structures of 54-4H03 from clonotype A in complex with A/California/04/2009 (H1N1) HA and 54-1G05 from clonotype B in complex with A/Solomon Island/3/2006 (H1N1) HA were determined to 3.5 and 4.2 Å resolutions, respectively (Table S2). Both 54-4H03 and 54-1G05 bind to the HA stem in a very similar orientation (Figure 2A). This orientation was previously observed in the V_H_6-1/D_H_3-3-encoded HA stem-directed bnAbs 56.a.09 (Joyce et al., 2016) and MEDI8852 (Kallewaard et al., 2016). Of note, 56.a.09 also utilized the same V_L_ gene segment V_κ_3-20 as 54-4H03 and 54-1G05, whereas MEDI8852 utilized the V_L_ gene segment V_κ_1-39. As expected from the similarity of germline origins, the epitopes of 54-4H03, 54-1G05, 56.a.09, and MEDI8852 are highly similar (Figures 2B and S2). Overall, despite the difference in HA specificity between different V_H_6-1-encoded HA stem-directed antibody clonotypes, their angles of approach and orientations are generally similar.

### 54-4H03 and 54-1G05 Have Different CDR H3 Conformations

**Figure 3:**
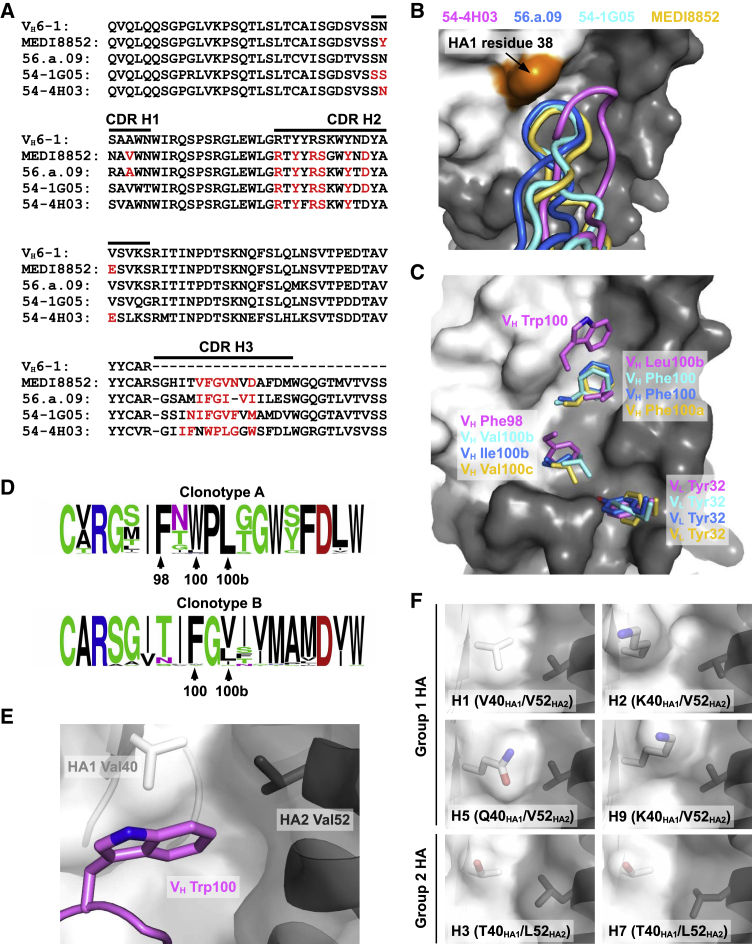
Comparison of the HA-Binding Modes among Different V_H_6-1-Encoded bnAbs (A) Alignment of the germline V_H_6-1 sequence with those of 54-4H03, 54-1G05, 56.a.09 (Joyce et al., 2016), and MEDI8852 (Kallewaard et al., 2016). The regions that correspond to CDR H1, H2, and H3 (Kabat numbering scheme) are indicated. Paratope residues are highlighted in red. (B) Conformations of CDR H3 from 54-4H03, 54-1G05, 56.a.09 (PDB 5K9K) (Joyce et al., 2016), and MEDI8852 (PDB 5JW4) (Kallewaard et al., 2016) are compared. HA1 surface is colored in white, HA2 in gray, and HA1 residue 38 in orange. (C) Residues on CDR H3 of 54-4H03, 54-1G05, MEDI8852 (PDB 5JW4) (Kallewaard et al., 2016), and 56.a.09 (PDB 5K9K) (Joyce et al., 2016) that are important for binding to the HA stem are shown. V_L_ Tyr32 on CDR L1 that is also important for binding is shown. The HA1 surface is colored in white and HA2 in gray. (D) Sequence variation of CDR H3 among members of clonotype A (including 54-4H03) and clonotype (including 54-1G05) are shown as sequence logos. The relative sizes of the letters represent their occurrence frequency. (E) Interaction between V_H_ Trp100 on CDR H3 of 54-4H03 with HA of A/California/04/2009 (H1N1) is highlighted. (F) Shapes of the binding pocket for accommodating V_H_ Trp100 of 54-4H03 among different HA subtypes are compared. H1 HA: PDB 3LZG (Xu et al., 2010a). H2 HA: PDB 3KU5 (Xu et al., 2010b). H3 HA: PDB 4FNK (Ekiert et al., 2012). H5 HA: PDB 4BGW (Xiong et al., 2013). H7 HA: PDB 4LN6 (Yang et al., 2013). H9 HA: PDB 1JSD (Ha et al., 2002).

We next wanted to compare the paratopes of 54-4H03, 54-1G05, 56.a.09, and MEDI8852. Many residues on the heavy chain (Figure 3A) and light chain (Figure S3) are commonly utilized for binding among 54-4H03, 54-1G05, 56.a.09, and MEDI8852. Except for CDR L2, all CDRs in 54-4H03, 54-1G05, 56.a.09, and MEDI8852 are involved in HA interactions (Figure S4). Most of the differences in the paratope sequences can be attributed to CDR H3 and CDR L3. Interestingly, despite the difference in the amino-acid sequences of CDR H3 in 54-1G05, 56.a.09, and MEDI8852, their CDR H3 conformations are relatively similar (Figures 3B and 3C). In contrast, the conformation of CDR H3 in 54-4H03 with a group 2-specific ancestor is distinct from 54-1G05, 56.a.09, and MEDI8852 with group 1-specific ancestors (Figure 3B). Compared to the CDR H3s of 54-1G05, 56.a.09, and MEDI8852, CDR H3 of 54-4H03 is further from HA1 residue 38 (Figure 3B). A highly conserved N-glycosylation at HA1 residue 38 in group 2 HA, but not in group 1 HA, can restrict the ability of certain HA stem-directed bnAbs to cross-react with group 2 influenza subtypes (Wu and Wilson, 2017). The N-glycosylation at HA1 residue 38 may impose a larger steric hindrance to the binding of 54-1G05, 56.a.09, and MEDI8852 than to 54-4H03, which preferentially binds group 2 HA. As a result, we conclude that the conformation of CDR H3 plays a substantial role in determining the specificity of V_H_6-1-encoded HA stem-directed bnAbs.

### HA-Binding Modes of CDR H3 in 54-4H03 and 54-1G05

To better understand how CDR H3 of 54-4H03 binds to the HA compared with 54-1G05 and 56.a.09, as well as MEDI8852, we looked more closely at specific CDR H3 residues interacting with the HA stem and found substantial differences. Three pockets in the HA stem are commonly targeted by 54-4H03, 54-1G05, 56.a.09, and MEDI8852 (Figure 3C). These include two pockets that are targeted by CDR H3 and one pocket that is targeted by Tyr32 in the light chains of 54-4H03, 54-1G05, 56.a.09, and MEDI8852 (Figure 3C). Of the two pockets commonly targeted by CDR H3, one pocket is filled by Phe98 in 54-4H03, whereas the other clonotypes insert aliphatic residues into this pocket (Val100b in 54-1G05, Ile100b in 56.a.09, and Val100c in MEDI8852). The other CDR H3 pocket is targeted by Leu100b in 54-4H03, but Phe occupies this pocket in 54-1G05, 56.a.09, and MEDI8852. In addition, in the upper part of the HA stem domain, another pocket is targeted by Trp100 in 54-4H03 but not by 54-1G05, 56.a.09, and MEDI8852. Sequence conservation among members in clonotype A and clonotype B indicates that the CDR H3 residues described above are highly selected for HA binding to 54-4H03 and 54-1G05, respectively (Figure 3D).

These differences in CDR H3 binding to HA stem pockets impact HA subtype specificity between the two clonotypes. The pocket in the upper part of the HA stem domain targeted only by Trp100 in 54-4H03 contains HA1 residue 40 and HA2 residue 52 (Figure 3E). Sequence variation can be observed for both residues among different HA subtypes (Zost et al., 2019). H3 and H7 from group 2 HA both have Thr and Leu at HA1 residue 40 and HA2 residue 52, whereas group 1 HAs, H1, H2, H5, and H9 have Val at HA2 residue 52, but different amino-acid variants at HA1 residue 40. At residue 40, H1 has a Val, while H2 and H9 have Lys, and H5 has a Gln. The pocket created by Val40_HA1_/Val52_HA2_ in H1 HA has a very similar shape to that formed by Thr40_HA1_/Leu52_HA2_ in H3 and H7 (Figure 3F), due to the side-chain similarity of Val40_HA1_ and Thr40_HA1_, and Val52_HA2_ and Leu52_HA2_. In contrast, the bulkier side chain of Gln40_HA1_ in H5 and Lys40_HA1_ in H2 and H9 may impose some steric hindrance for binding of 54-4H03 to this pocket (Figure 3F). These structural findings are consistent with the weaker reactivity of 54-4H03 to these group 1 subtype HAs.

**Figure 4:**
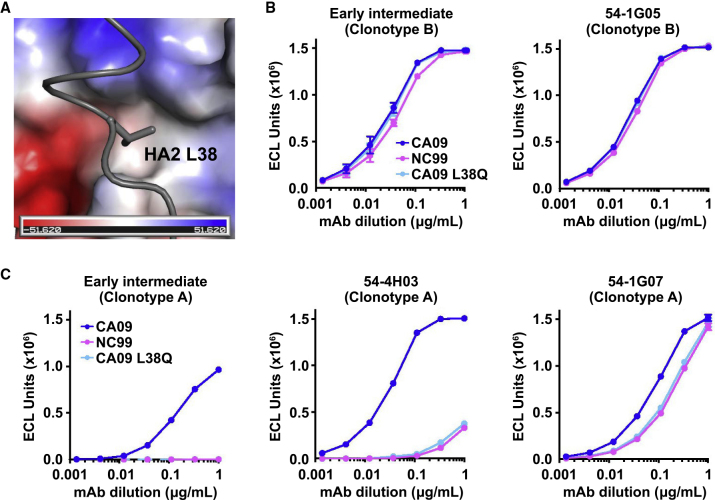
Amino-Acid Variation in Residue 38 of H1 HA Determines Reactivity to Clonotype A but not Clonotype B (A) Interaction of 54-4H03 with HA2 L38 in CA09 HA is shown. HA2 L38 is shown in stick representation and 54-4H03 in a surface electrostatic representation. (B and C) (B) Binding curves of early intermediate and 54-1G05 from clonotype B, and (C) early intermediate, 54-4H03, 54-1G07 from clonotype A to CA09, NC99, and CA09 L38Q mutant HAs as measured by meso scale discovery (MSD). Antibodies were in IgG format.

Other structural elements may also play a role in determining the breadth of clonotype A. For example, while H1N1 strains A/California/04/2009 (CA09) and A/New Caledonia/20/1999 (NC99) both have Val40_HA1_/Val52_HA2_, 54-4H03 more strongly neutralizes CA09 than NC99 (Figure 1C). Structural analysis revealed that CA09 has a Leu at HA2 residue 38, which interacts with a hydrophobic pocket in 54-4H03 (Figure 4A). In comparison, H1 NC99 has a Gln at this residue, which would not favor interaction with the hydrophobic pocket. Indeed, while substitution of Gln for Leu at HA2 residue 38 of CA09 (CA09 L38Q) had no effect on clonotype B binding (Figure 4B), it drastically diminished the ability of clonotype A to bind to CA09 (Figure 4C). Binding of clonotype A members to the CA09 L38Q mutant was similar to that to H1 NC99 (Figure 4C). These analyses reveal key differences in the stem regions of group 1 HA subtypes that can have a significant impact on the breadth of HA stem-directed mAbs.

### Different Binding Modes of D_H_3–3-Encoded Phe

**Figure 5:**
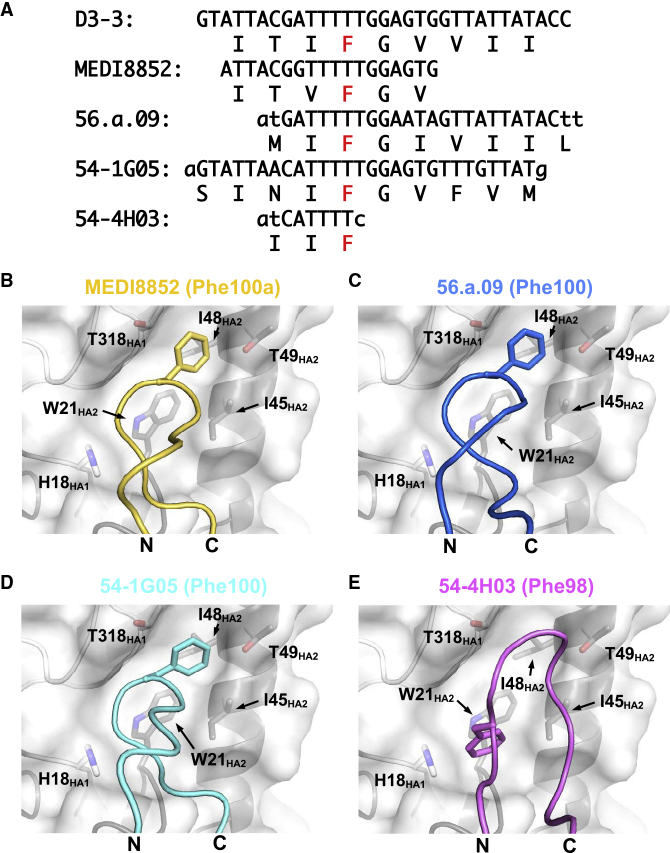
D_H_3–3-Encoded Phe of Clonotypes A and B Target Different Regions of the HA Stem (A) The D_H_ gene regions of MEDI8852, 56.a.09, 54-1G05 (clonotype B), and 54-4H03 (clonotype A) are aligned to the D3-3^∗^01 germline gene. For each antibody, the amino-acid sequence is shown below the nucleotide sequence. The amino-acid sequence from the frame 3 translation is shown for the D3-3^∗^01 germline gene. The Phe of interest is highlighted in red. Nucleotides from N-region additions are shown in lower case. (B–E) CDR H3 loops of (B) MEDI8852, (C) 56.a.09, (D) 54-1G05, and (E) 54-4H03 are shown. The side chain of D_H_3-3-encoded Phe is shown in stick representation. HA is shown as a semi-transparent surface with superimposed tube representation for the backbone and Phe side chain of interest in stick representation. The N- and C-termini of the CDR H3 loops are indicated as “N” and “C,” respectively. For each antibody, the Kabat sequence number of the Phe of interest is indicated in parentheses.

We also observed differences in binding of residues that are conserved between 54-4H03 and the three other bnAbs, 54-1G05, MEDI8852 (Kallewaard et al., 2016), and 56.a.09 (Joyce et al., 2016). All four bnAbs utilize translation frame 3 of the D_H_3–3 gene segment in their CDR H3. This translation frame contains a Phe (Figure 5A), which is retained in the CDR H3s of 54-4H03, 54-1G05, MEDI8852, and 56.a.09 but, due to differences in its register in CDR H3, is annotated as a different residue number in different antibodies. This residue is Phe98 in 54-4H03, Phe100 in both 54-1G05 and 56.a.09, and Phe100a in MEDI8852. The D_H_3–3-encoded Phe in 54-1G05, MEDI8852, and 56.a.09 targets a pocket that is formed by HA1 residue 318 and HA2 residues 48 and 49 (Figures 5B–5D). Interestingly, two non-V_H_6-1-encoded HA stem-directed bnAbs 39.29 (Nakamura et al., 2013) and 429 B01 (Matsuda et al., 2019) also utilize the D_H_3–3-encoded Phe to target this same pocket (Matsuda et al., 2019). In contrast, the D_H_3–3-encoded Phe in 54-4H03 targets a pocket that is lower down the HA stem and is formed by HA1 residue 18 and HA2 residues 21 and 45 (Figure 5E). As a result, bnAbs can utilize residues encoded by the exact same codons in the D_H_3–3 gene segment to target different regions of the HA stem.

### Somatic Mutations Enhance the Breadth of Clonotype A

**Figure 6:**
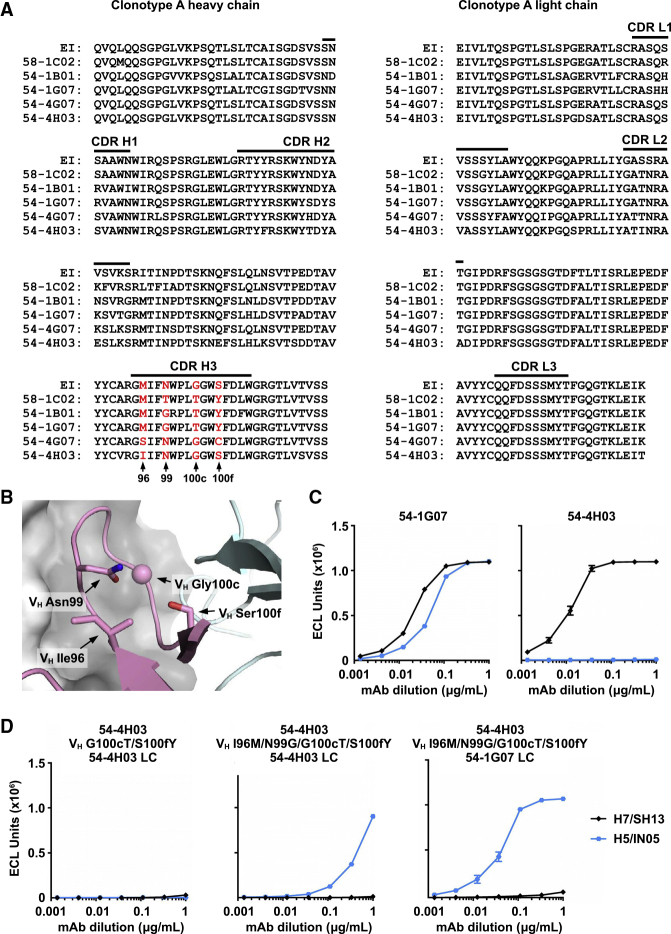
Analysis of Somatic Mutations in Clonotype A (A) Sequence alignment of the heavy and light chains of representative antibodies from clonotype A are shown. Residues of interest are highlighted in red with their positions indicated. EI, early intermediate. (B) Residues of interest are shown on the crystal structure. HA surface is colored in gray, Fab heavy chain in pink, and light chain in cyan. The side chains of residues of interest are shown in stick representation. Since Gly100c does not have a side chain, the Cα of Gly100c is shown as a sphere. (C) Binding curves of 54-1G07, 54-4H03, and (D) different 54-4H03 heavy-chain mutants paired with either the 54-4H03 light chain or the 54-1G07 light chain to H5 and H7 HAs as measured by meso scale discovery (MSD). Antibodies were in IgG format.

To obtain insight into how cross-group reactivity is developed in clonotype A, we investigated how it acquired binding to group 1 subtype H5 HA. Among members of clonotype A, 58-1C02, 54-1B01, and 54-1G07 interact strongly with group 1 subtype H5 HA (Figure 1A), whereas 54-4G07 and 54-4H03 do not. We therefore focused on the differences between 54-1G07 and 54-4H03. Due to somatic mutations, the CDR H3 sequences of 54-1G07 and 54-4H03 differ by four amino-acid residues. Specifically, 54-1G07 has three somatic mutations N99G, G100cT, and S100fY on CDR H3, whereas 54-4H03 has only one somatic mutation M96I on CDR H3 (Figures 6A and 6B).

Interestingly, introduction of the two mutations V_H_ G100cT/S100fY into 54-4H03 abolished binding to H7 HA without gain of binding to group 1 subtype H5 HA (Figures 6C and 6D). We further introduced two additional V_H_ mutations I96M/N99G, such that the CDR H3 sequence matched that of 54-1G07. Binding of the 54-4H03 V_H_ I96M/N99G/G100cT/S100fY mutant to group 1 subtype H5 HA was now observed, although not as strongly as 54-1G07 (Figure 6D). These results indicate that somatic mutations in CDR H3 are important for enhancing the breadth of clonotype A to group 1 HA. Somatic mutations G100cT and S100fY on CDR H3 would stabilize the conformation of CDR H3. Specifically, mutation of Gly100c to Thr100c would help to rigidify CDR H3, whereas mutation of Ser100f to Tyr100f may acquire a π–π stacking interaction with Tyr49 of the light chain (Figure S5). Residues 100c and 100f are both too distant to interact directly with HA. Consistently, most somatic mutations in 54-4H03 do not contact HA (Figure S6), indicating that the affinity maturation pathway of clonotype A is largely driven by non-paratope residues that stabilize the CDR H3 conformation.

When 54-4H03 V_H_ I96M/N99G/G100cT/S100fY is paired with the light chain from 54-1G07, its binding to group 1 subtype H5 HA is as strong as that of 54-1G07 (Figure 6D). This observation implies that somatic mutations on the light chain are also important for enhancing the breadth of clonotype A to group 1 HA. Nevertheless, none of the 54-4H03 mutants were able to bind group 2 subtype H7 HA, regardless of light chain identity, suggesting that additional somatic mutations in clonotype A are involved in maintaining binding to group 2 HA when acquiring breadth to group 1 HA.

### Somatic Mutations Enhance the Breadth of Clonotype B

**Figure 7:**
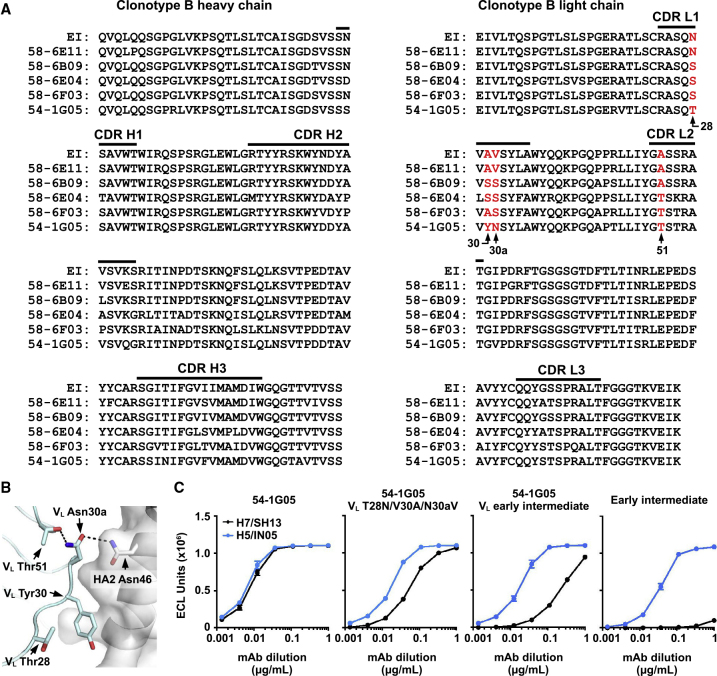
Analysis of Somatic Mutations in Clonotype B (A) Sequence alignment of the heavy and light chains of representative antibodies from clonotype B are shown. Residues of interest are highlighted in red with their positions indicated. EI: early intermediate. (B) Residues of interest are shown on the structure. The HA surface is colored in gray, and Fab light chain in cyan. The side chains of residues of interest are shown in stick representation. (C) Binding curves of the early intermediate of clonotype B, 54-1G05, and different 54-1G05 mutants to H5 and H7 HAs as measured by meso scale discovery (MSD). Antibodies were in IgG format.

We also analyzed the structural impact of somatic mutations in 54-1G05, the broadest member of clonotype B, to understand how cross-group reactivity developed in clonotype B. The somatic mutations on CDR L1 appear to be important for the development of cross-group reactivity. CDR L1 of 54-1G05 contains somatic mutations V_L_ N28T, A30Y, and V30aN, which are not observed in other analyzed members of clonotype B (Figures 7A and 7B). V_L_ A30Y contributes 120 Å^2^ buried surface area on 54-1G05 upon interaction with HA (Figure S6). Other somatic mutations in 54-1G05 also help stabilize CDR L1. For example, the side chain of somatic mutant V_L_ A51T forms hydrogen bonds with the main chain and side chain of somatic mutant V_L_ V30aN, which in turn forms a side chain-side chain hydrogen bond with HA2 Asn46 (Figure 7B). Consistent with the structural conclusions, introduction of mutations V_L_ T28N/Y30A/N30aV into 54-1G05, which reverts its CDR L1 residues to the early intermediate sequence, strongly decreased binding to group 2 subtype H7 HA and mildly decreased binding to group 1 subtype H5 HA (Figure 7C). When 54-1G05 V_H_ is paired with V_L_ from the early intermediate of clonotype B (54-1G05 V_L_ early intermediate), binding to group 2 subtype H7 HA was further decreased (Figure 7C). However, binding of the 54-1G05 V_L_ early intermediate to group 2 H7 HA was still much stronger than that of the full V_H_ and V_L_ early intermediate, indicating that somatic mutations in the heavy chain are also important for enhancing the breadth of clonotype B to group 2 HA.

In summary, during affinity maturation of the V_H_6-1-encoded influenza HA stem-directed bnAbs, a wide range of strategies are employed, including stabilization of the CDR loops, increasing the interaction surface area, and acquiring additional hydrogen bonds to the HA.

## Discussion

Influenza HA stem-directed bnAbs were only discovered in humans just over a decade ago (Kashyap et al., 2008; Throsby et al., 2008), and subsequent studies have shown that certain antibody germline genes can give rise to HA stem-directed bnAbs across multiple donors (Andrews and McDermott, 2018). These bnAbs include V_H_1-69 (Dreyfus et al., 2012; Ekiert et al., 2009; Kashyap et al., 2008; Lang et al., 2017; Nakamura et al., 2013; Sui et al., 2009; Throsby et al., 2008; Wrammert et al., 2011; Yamayoshi et al., 2018), V_H_1-18 (Andrews et al., 2017; Ekiert et al., 2011; Nakamura et al., 2013; Wu et al., 2015), V_H_3-30 (Corti et al., 2011; Fu et al., 2016; Nakamura et al., 2013; Wyrzucki et al., 2014), V_H_6-1 (Andrews et al., 2017; Joyce et al., 2016; Kallewaard et al., 2016), and D_H_3-9 (Wu et al., 2018). Some classes of bnAbs, such as V_H_1-18 bnAbs with a Q-x-x-V motif in CDR H3, initially bind group 2 HA subtypes and then gain reactivity to group 1 with somatic mutations (Joyce et al., 2016; Wu et al., 2015). Others, such as V_H_6-1-encoded bNAbs, preferentially bind group 1 subtypes and then increase in breadth to group 2 subtypes, similar to the V_H_6-1 clonotype B described here (Joyce et al., 2016; Kallewaard et al., 2016). An important finding of our work, however, is that cross-group V_H_6-1-encoded bnAbs can also develop from group 2-specific germline precursors. We describe V_H_6-1 clonotype A, which started as a largely group 2-specific clonotype and then gained cross-reactivity to group 1 through somatic mutations. V_H_6-1-encoded bnAbs that are derived from group 2-specific germline precursors are unlikely to be rare since they could also be observed in at least one other donor after H7N9 vaccination (Figure S7).

The epitopes of clonotype A and those of other V_H_6-1-encoded bnAbs that are derived from group 1-specific germline precursors largely overlap. As illustrated by our structural analysis, the specificity of V_H_6-1-encoded bnAbs appears to be influenced by the conformation of CDR H3 in addition to individual amino-acid differences. These observations imply that VDJ recombination, including N-nucleotide additions and J gene usage, plays a critical role in determining the germline specificity of V_H_6-1-encoded HA stem-directed antibodies. Although the CDR H3s in clonotype A and clonotype B have very different conformations, they are able to recognize similar hydrophobic pockets in the HA stem. This convergent structural feature is likely the key for the development of cross-group bnAbs since those hydrophobic pockets are commonly targeted by other cross-group HA stem-directed bnAbs that are encoded by diverse germlines with very different overall binding modes (Wu and Wilson, 2019). At the same time, the CDR H2 on V_H_6-1-encoded HA stem-directed antibodies is also important, as indicated by its large buried surface area upon binding (Figure S4). Most paratope residues on CDR H2 are encoded by the germline (Figure 3A), which may largely explain why V_H_6-1 is commonly elicited to target the HA stem. Overall, V_H_6-1 provides a versatile framework for evolving HA stem-directed bnAbs, starting from a germline precursor with either group 1 or group 2 specificity.

Another important finding in this study is the co-existence of two V_H_6-1 clonotypes in a single donor. This donor was immunized with an H5N1 vaccine and then with an H7N9 vaccine 5 years later (Andrews et al., 2017). Members of both clonotypes could be found in the memory B cell population before the H5N1 vaccine, and an expansion of both clonotypes was observed after both H5N1 and H7N9 vaccination (Andrews et al., 2017). As expected given their HA group preferences, clonotype B was more prevalent after group 1 H5N1 vaccination, and clonotype A dominated after group 2 H7N9 immunization. Whether vaccination with H5N1 and H7N9 leads to greater breadth of either clonotype or simply restimulated cross-reactive clonotype members developed previously from natural infection or seasonal vaccination is unclear from phylogenetic analysis. From a vaccinology perspective, identification of both clonotypes in the same individual supports the idea that vaccination with both group 1 and group 2 immunogens can facilitate the evolution of antibody breadth with the potential to acquire influenza A cross-group reactivity.

## STAR★Methods

### Key Resources Table

**Table:** 

REAGENT or RESOURCE	SOURCE	IDENTIFIER
ExpiCHO Expression System Kit	Thermo Fisher Scientific	A29133
Expi293 Expression System Kit	ThermoFisher	Cat# A14635
HyClone insect cell culture medium	GE Healthcare	SH30280.03
Phosphate-buffered saline (PBS)	Thermo Fisher Scientific	14040133
Ni-NTA Superflow	Qiagen	30450
HA and NA protein sequences GISAID: H7N9 SH13 and H6N1 TW13; NCBI influenza virus database: H3N2 HK68, H3N2 TX12, H10N8 JX13, H1N1 CA09, H1N1 NC99, H5N1 IN05, H2N2 SI57 and H9N2 HK99	GISAID; http://gisaid.org	N/A
	NCBI Influenza virus database; https://www.ncbi.nlm.nih.gov/genomes/FLU/	N/A
DH10Bac competent cells	Thermo Fisher Scientific	10361012
Sulfo-tag anti-human IgG	Meso Scale Discovery	Cat# R32AJ-1
Protein A Sepharose	GE LifeSciences	Cat# 17-1279-03

**Chemicals and Recombinant Proteins**		

DpnI	New England Biolabs	R0176L
Trypsin	New England Biolabs	P8101S
Fugene 6 Transfection Regent	Promega	E2691
Luciferase Assay System	Promega	E1501
Sodium chloride (NaCl)	Sigma-Aldrich	S9888
Tris Base	Sigma-Aldrich	11814273001
Concentrated hydrochloric acid (HCl)	Sigma-Aldrich	H1758
Sodium azide (NaN_3_)	Sigma-Aldrich	S2002
Bovine Serum Albumin (BSA)	Sigma-Aldrich	A9418
Tween 20	Fisher Scientific	BP337-500
Chemicals for protein crystallization	Hampton Research	N/A

**Critical Commercial Assays**		

In-Fusion HD Cloning Kit	Takara	639647
KOD Hot Start DNA Polymerase	EMD Millipore	71086-3
PCR Clean-Up and Gel Extraction Kit	Clontech Laboratories	740609.250
QIAprep Spin Miniprep Kit	Qiagen	27106
NucleoBond Xtra Maxi	Clontech Laboratories	740414.100
AviTag Kit	Avidity	Cat# BirA-500

**Deposited Data**		

X-ray coordinates and structure factors	This manuscript	PDB: 6WIY, 6WIZ, 6WJ0, 6WJ1

**Cell Lines**		

293A cells	Thermo Fisher Scientific	R70507
ExpiCHO cells	Thermo Fisher Scientific	A29127
Sf9 cells	ATCC	CRL-1711
High Five cells	Thermo Fisher Scientific	B85502
Expi293F cells	Thermo Fisher Scientific	Cat# A14527

**Oligonucleotides**		

Forward primer for heavy chain cloning: 5’-CCT GGC TCT ACC GGA CAA GTA CAA TTG CAG CAA TCT GGC-3’	Integrated DNA Technologies	N/A
Reverse primer for 54-4H03 heavy chain cloning: 5’-GCC CTT TGT GCT GGG AGA AGA TAC AGA AAC CAA CGT GCC-3’	Integrated DNA Technologies	N/A
Reverse primer for 54-1G05 heavy chain cloning: 5’-GCC CTT TGT GCT GGG TGA ACT TAC TGT AAC GGC TGT GCC-3’	Integrated DNA Technologies	N/A
Forward primer for light chain cloning: 5’-CCC GGC AGC ACC GGC GAA ATT GTG TTG ACT CAG AGT CCG-3’	Integrated DNA Technologies	N/A
Reverse primer for 54-4H03 light chain cloning: 5’-TGC TGC CAC GGT CCT AGT AAT TTC CAG CTT TGT CCC TTG-3’	Integrated DNA Technologies	N/A
Reverse primer for 54-1G05 light chain cloning: 5’-TGC TGC CAC GGT CCT TTT AAT TTC GAC TTT CGT CCC ACC-3’	Integrated DNA Technologies	N/A

**Recombinant DNA**		

phCMV3-54-4H03 Fab heavy chain	This manuscript	N/A
phCMV3-54-4H03 light chain	This manuscript	N/A
phCMV3-54-1G05 Fab heavy chain	This manuscript	N/A
phCMV3-54-1G05 light chain	This manuscript	N/A
pFastBac-A/Solomon Islands/3/2006 (H1 HA)	(Ekiert et al., 2012)	N/A
pFastBac-A/California/04/2009 (H1 HA)	(Ekiert et al., 2012)	N/A
pCMV/R-HA-AviHis	(Andrews et al., 2017)	N/A
pCMV/R IgG heavy chain expression vector	(Andrews et al., 2017)	N/A
pCMV/R Kappa heavy chain expression vector	(Andrews et al., 2017)	N/A

**Software and Algorithms**		

HKL2000	(Otwinowski and Minor, 1997)	N/A
Phaser	(McCoy et al., 2007)	N/A
Coot	(Emsley et al., 2010)	N/A
Refmac5	(Murshudov et al., 2011)	N/A
MolProbity	(Chen et al., 2010)	N/A
Prism 7/8	Graphpad	http://www.graphpad.com
Seaview		Doua.prabi.fr/software/seaview RRID: SCR_015059
Dendroscope	Huson and Scornavacca, 2012	Dendroscope.org
Octet analysis software 9.0	Fortebio	http://www.moleculardevices.com

**Other**		

Streptavidin biosensors	ForteBio	Cat# 18-5019
Streptavidin coated 384-well plates	Meso Scale Discovery	Cat# L25SA-5

### Resource Availability

#### Lead Contact

Further information and requests for resources and reagents should be directed to and will be fulfilled by the Lead Contact, Ian A. Wilson (wilson@scripps.edu).

#### Materials Availability

Plasmids generated from this study will be available upon request.

#### Data and Code Availability

The X-ray coordinates and structure factors have been deposited in the RCSB Protein Data Bank under accession codes 6WIY, 6WIZ, 6WJ0, and 6WJ1.

### Experimental Model and Subject Details

#### Cell Cultures

ExpiCHO cells were maintained according to the manufacturer’s instructions (Thermo Fisher Scientific). Sf9 cells (*Spodoptera frugiperda* ovarian cells, female) and High Five cells (*Trichoplusia ni* ovarian cells, female) were maintained HyClone insect cell culture medium.

### Method Details

#### Isolation and Production of HA-Specific bnAbs

Immunoglobulin heavy and light chains were PCR amplified and sequenced from single cell sorted HA-specific B cells from one subject (identified as Volunteer #2), who was immunized with both H5N1 and H7N9 vaccines in two vaccine trials 5 years apart as previously described (Andrews et al., 2017). Clonotypes A and B were formerly referred to as lineages 2 and 3 (Andrews et al., 2017). To produce antibodies recombinantly, Expi293 cells were transfected with plasmids encoding Ig heavy and light chain pairs with ExpiFectamine (Thermo Fisher Scientific). Monoclonal antibodies were purified from the cell supernatant using sepharose Protein A (Pierce).

#### Phylogenetic Analysis

Phylogenetic analysis of Immunoglobulin heavy chains was performed using the Maximum-Likelihood PhyML algorithm (Guindon et al., 2010). Trees were displayed using Dendroscope rooted on the germline V_H_6-1 gene (Huson and Scornavacca, 2012). Inferred early intermediates were determined as previously described (Corbett et al., 2019). Briefly, a maximum-likelihood phylogenetic tree was constructed from heavy and light chain sequences and the most recent common ancestor (MRCA) of the lineage was inferred. Sequences of early intermediates were composed of the germline V gene and the inferred MRCA CDR3.

#### HA Antibody Binding Assay

Meso Scale Discovery (MSD) 384 well streptavidin-coated SECTOR® Imager 2400 Reader Plates were blocked with 5% MSD Blocker A for 60 minutes and washed six times with wash buffer (PBS+0.05% Tween). Plates were then coated with biotinylated HA protein for one hour and washed. Monoclonal antibodies were diluted in 1% MSD Blocker A to 1 μg/ml, serially diluted 3-fold, and added to the coated plates. After one-hour incubation, plates were washed and incubated with SULFO-TAG conjugated anti-human IgG for one hour. After washing, the plates were read using 1X MSD Read Buffer using an MSD SECTOR® Imager 2400. Binding curves were plotted and the area under the curve (AUC) was determined using Prism 8. HAs from the following strains were tested: H7N9 A/Shanghai/02/2013, H3N2 A/Texas/50/2012, H3N2 A/Hongkong/1/1968, H10N8 A/Jiangxi-Donghu/346/2013, H1N1 A/California/04/2009, H1N1 A/New Caledonia/20/1999, H5N1 A/Indonesia/05/2005, H2N2 A/Singapore/2/1957, H9N2 A/Hongkong/1073/1999, and H6N1 A/Taiwan/2/2013.

#### Kinetics Evaluation Using Biolayer Interferometry (BLI)

All kinetics evaluation and analyses were performed using Octet Red384 (Pall FortéBio) and accompanying software version 9.0. Biotinylated HA protein (at 5 μg/ml) was loaded onto streptavidin-coated biosensors, which had been previously equilibrated in assay buffer. Biosensors were then transferred to wells containing assay diluent to remove unbound protein and establish baseline signal. After the baseline had been established, the biosensors complexed with HA protein were allowed to associate with different concentrations of Fab (3200 nM, 800 nM, 200 nM and 50 nM). After 180 seconds of association, the fully complexed biosensors were transferred back to the baseline wells and the dissociation was measured for 300 seconds. Raw binding data were analyzed in the Octet software using a global fit, alignment to baseline and inter-step correction to dissociation. In this way, each HA-Fab combination was assigned the following kinetic analysis measurements: K_*D*_, K_*on*_, and K_*off*_. In cases where no measurable dissociation was detected, the K_*D*_ is listed as “ND”. In cases where the highest measurable response was less than 0.5 nm and the R^2^ was less than 0.9, the HA-Fab pair was noted as “No Binding Observed”.

#### Pseudotype Neutralization Assay

Influenza HA-NA pseudotyped lentiviruses that harbor a luciferase reporter gene were produced as described previously (Naldini et al., 1996; Yang et al., 2007). Pseudovirus was produced by transfection of 293T cells of HA and corresponding NA along with the lentiviral packaging and reporter plasmids. For H1N1, H2N2, H3N2, H7N9 and H9N2 pseudoviruses, a human type II transmembrane serine protease TMPRSS2 gene was also cotransfected for proteolytic activation of HA to HA1/HA2. Forty-eight hours after transfection, supernatants were harvested, filtered and frozen.

Neutralization assays were performed as described previously (Joyce et al., 2016). Briefly, pseudovirus was mixed with various dilutions of monoclonal antibodies for 45 minutes followed by addition to 293A cells (Thermo Fisher Scientific) in 96-well plates. Three days after infection, cells were lysed, and luciferase assay reagent was added to measure luciferase activity. The following pseudovirus were tested: H7N9 A/Shanghai/01/2013, H3N2 A/Texas/50/2012, H3N2 A/Hongkong/1/1968, H10N8 A/Jiangxi/IPB13/2013, H1N1 A/California/04/2009, H1N1 A/New Caledonia/20/1999, H5N1 A/Indonesia/05/2005, H2N2 A/Singapore/1/1957, H9N2 A/Hongkong/1074/1999, and H6N1 A/Taiwan/2/2013.

#### Fab Expression and Purification for Crystallization

The heavy chains and light chains of 54-4H03 and 54-1G05 were cloned into a phCMV3 vector. The heavy chain and light chain plasmids were co-transfected into ExpiCHO cells (Thermo Fisher Scientific) at 2:1 molar ratio (heavy to light) using the Max titer protocol as described by the manufacturer’s instructions for the ExpiCHO Expression System. Fabs were purified from the supernatant by a 5 mL HiTrap Protein G HP antibody purification column ÄKTAxpress (GE Healthcare) and subsequently by size exclusion chromatography on a Hiload 16/90 Superdex 200 column (GE Healthcare) in 20 mM Tris pH 8.0, 150 mM NaCl, and 0.02% NaN_3_. For crystallization of the apo forms, Fabs were further buffer exchanged into 10 mM Tris pH 8.0, 50 mM NaCl, and 0.02% NaN_3_, and concentrated to 10 mg mL^-1^.

#### HA Expression and Purification for Crystallization

Briefly, the HA ectodomains (HA1 residues 11-329 and HA2 residues 1-176, based on H3 numbering) of A/California/04/2009 (H1N1) and A/Solomon Island/3/2006 (H1N1) were fused with an N-terminal gp67 signal peptide and a C-terminal BirA biotinylation site, thrombin cleavage site, trimerization domain, and His_6_ tag, and then cloned into a customized baculovirus transfer vector (Ekiert et al., 2011). Recombinant bacmid DNA was generated using the Bac-to-Bac system (Life Technologies). Baculovirus was generated by transfecting purified bacmid DNA into Sf9 cells using FuGene HD (Promega). HA was expressed by infecting suspension cultures of High Five cells (Life Technologies) with baculovirus at an MOI of 5 to 10 and incubating at 28°C shaking at 110 rpm for 72 hours. The supernatant was concentrated. HA0 was purified by Ni-NTA, and buffer exchanged into 20 mM Tris-HCl pH 8.0 and 150 mM NaCl. The HA0 was then treated with trypsin (New England Biolabs) to remove the C-terminal tag (BirA biotinylation site, thrombin cleavage site, trimerization domain, and His_6_ tag) and to produce the cleaved mature HA (HA1/HA2). The trypsin-digested HA was then purified by size exclusion chromatography on a Hiload 16/90 Superdex 200 column (GE Healthcare) in 20 mM Tris pH 8.0, 150 mM NaCl, and 0.02% NaN_3_.

#### Fab-HA Complex Formation for Crystallization

54-4H03 Fab or 54-1G05 Fab was incubated with the purified HA trimer in a molar ratio of 4.5:1 overnight at 4°C. The Fab-HA complexes were purified by size exclusion chromatography on a Hiload 16/90 Superdex 200 column (GE Healthcare) in 10 mM Tris pH 8.0, 50 mM NaCl, and 0.02% NaN_3_, and concentrated to 10 mg mL^-1^.

#### Crystallization and Structural Determination

Crystal screening was carried out using our high-throughput, robotic CrystalMation system (Rigaku) using the sitting drop vapor diffusion method at 4°C and 20°C with each drop consisting of 100 nL protein + 100 nL precipitant. Diffraction-quality crystals for 54-4H03 Fab apo form and 54-1G05 Fab apo form were both obtained in 20% PEG 8000, 0.2 M NaCl, and 0.1 M phosphate-citrate pH 4.2 at 20°C. Diffraction-quality crystals for 54-4H03 Fab in complex with A/California/04/2009 HA were obtained in 11% PEG 6000 and 0.1 M MES pH 5.6 at 20°C. Diffraction-quality crystals for 54-1G05 Fab in complex with A/Solomon Island/3/2006 HA were obtained in 50% PEG 200 and 0.1 M CHES pH 9.5 at 20°C. The crystals for apo forms of 54-4H03 Fab and 54-1G05 Fab were cryoprotected by soaking in well solution supplemented with 25% glycerol. The crystals for 54-4H03 Fab in complex with A/California/04/2009 were cryoprotected by soaking in well solution supplemented with 20% PEG 200. The crystals were flash cooled and stored in liquid nitrogen until data collection. Diffraction data were collected at the APS GM/CA-CAT 23ID-B and 23ID-D, and then indexed, integrated and scaled using HKL2000 (HKL Research, Charlottesville, VA) (Otwinowski and Minor, 1997). The structure was solved by molecular replacement using Phaser (McCoy et al., 2007), modeled using Coot (Emsley et al., 2010), and refined using Refmac5 (Murshudov et al., 2011), and PHENIX (Adams et al., 2010). For molecular replacement, PDB 4M4Y (Hong et al., 2013) was used as the model for A/California/04/2009 HA, PDB 6FYT (Laursen et al., 2018) for A/Solomon Island/3/2006 HA, and homology models generated by PIGSPro (Lepore et al., 2017) were used for MR of 54-4H03 and 54-1G05. Ramachandran statistics were calculated using MolProbity (Chen et al., 2010).

#### Buried Surface Area Calculation

Solvent accessibility was computed by DSSP (Kabsch and Sander, 1983). Buried surface area (BSA) was calculated by subtracting the solvent accessibility of the apo form by that of the bound form. HA and Fab residues that had a non-zero BSA were identified as epitope and paratope residues, respectively.

### Quantification and Statistical Analysis

Statistical analysis was not performed in this study.

## Supplemental Information

Document S1. Figures S1–S7 and Tables S1 and S2

Document S2. Article plus Supplemental Information
