# Supplementary material for: Convergent Evolution in Breadth of Two VH6-1-Encoded Influenza Antibody Clonotypes from a Single Donor
Source: Cell Host Microbe. 2020 Sep 9;28(3):434–444.e4. doi: 10.1016/j.chom.2020.06.003 (PMC7486241; doi:10.1016/j.chom.2020.06.003)
Supplement: Document S1. Figures S1–S7 and Tables S1 and S2 [file mmc1.pdf]

Cell Host & Microbe, Volume *28*

Supplemental Information

Convergent Evolution in Breadth of Two V_H6-1-Encoded Influenza Antibody Clonotypes from a Single Donor

Nicholas C. Wu, Sarah F. Andrews, Julie E. Raab, Sarah O'Connell, Chaim A. Schramm, Xintao Ding, Michael J. Chambers, Kwanyee Leung, Lingshu Wang, Yi Zhang, John R. Mascola, Daniel C. Douek, Julie E. Ledgerwood, Adrian B. McDermott, and Ian A. Wilson

Table S1, related to Figure 1. Binding of mAb Fabs to HA by biolayer interferometry (BLI)

HA	Fab	KD	Kon	Koff
H7 SH13	ClonA UCA	1.42E-06	2.61E+04	3.70E-02
	58-1C02	7.27E-06	6.51E+04	4.73E-01
	54-1B01	3.81E-06	1.22E+04	4.63E-02
	54-1G07	4.65E-06	1.37E+04	6.35E-02
	54-4G07	1.04E-06	2.22E+04	2.31E-02
	54-4H03	1.59E-07	2.55E+04	4.05E-03
	ClonB UCA	6.17E-06	2.09E+04	1.29E-01
	58-6E11	No Binding		
	58-6B09	No Binding		
	58-6E04	No Binding		
	58-6F03	1.43E-06	1.06E+04	1.53E-02
	54-1G05	9.61E-07	4.99E+04	4.79E-02
H3 HK68	ClonA UCA	4.31E-09	9.64E+04	4.15E-04
	58-1C02	2.80E-09	8.37E+04	2.35E-04
	54-1B01	NA	6.25E+04	No Diss
	54-1G07	NA	5.66E+04	No Diss
	54-4G07	4.94E-09	5.11E+04	2.53E-04
	54-4H03	NA	9.14E+04	No Diss
	ClonB UCA	7.59E-07	1.20E+04	9.13E-03
	58-6E11	6.43E-07	9.49E+03	6.11E-03
	58-6B09	3.11E-06	1.10E+04	3.43E-02
	58-6E04	5.90E-07	1.15E+04	6.81E-03
	58-6F03	4.36E-08	1.66E+05	7.25E-03
	54-1G05	1.37E-08	1.32E+05	1.80E-03
H3 TX12	ClonA UCA	6.96E-08	3.17E+04	2.21E-03
	58-1C02	4.24E-08	1.96E+04	8.33E-04
	54-1B01	5.72E-09	1.48E+04	8.45E-05
	54-1G07	4.28E-09	2.12E+04	9.06E-05
	54-4G07	6.29E-08	2.54E+04	1.60E-03
	54-4H03	2.27E-09	5.24E+04	1.19E-04
	ClonB UCA	No Binding		
	58-6E11	No Binding		
	58-6B09	No Binding		
	58-6E04	No Binding		
	58-6F03	7.54E-07	3.13E+04	2.36E-02
	54-1G05	3.56E-07	6.63E+04	2.36E-02
H10 JX13	ClonA UCA	2.90E-06	2.10E+04	6.10E-03
	58-1C02	No Binding		
	54-1B01	1.40E-06	1.90E+03	1.10E-02
	54-1G07	4.50E-06	1.20E+04	5.50E-02
	54-4G07	1.90E-06	2.30E+04	4.20E-02
	54-4H03	1.50E-07	6.40E-04	9.30E-03
	ClonB UCA	3.26E-06	3.37E+04	1.10E-01
	58-6E11	4.70E-06	7.80E+04	3.60E-01
	58-6B09	1.30E-06	1.00E+04	1.30E-02
	58-6E04	1.50E-06	8.70E+02	1.30E-02
	58-6F03	1.50E-07	6.40E+04	9.30E-03
	54-1G05	1.20E-06	2.30E+04	2.80E-02
H6 TW13	ClonA UCA	No Binding		
	58-1C02	3.65E-09	9.14E+03	3.33E-05
	54-1B01	4.21E-07	3.66E+03	1.54E-03
	54-1G07	2.56E-08	4.72E+03	1.21E-04
	54-4G07	2.74E-06	6.45E+03	1.77E-02
	54-4H03	5.35E-06	7.11E+03	3.80E-02
	ClonB UCA	2.34E-07	2.70E+04	6.33E-03
	58-6E11	1.60E-08	2.62E+04	4.18E-04
	58-6B09	2.23E-07	3.58E+04	7.97E-03
	58-6E04	NA	5.69E+04	No Diss
	58-6F03	NA	5.42E+04	No Diss
	54-1G05	5.09E-10	3.90E+04	1.99E-05

HA	Fab	KD	Kon	Koff
H1 CA09	ClonA UCA	1.35E-06	2.26E+04	3.04E-02
	58-1C02	NA	2.80E+04	No Diss
	54-1B01	NA	1.63E+04	No Diss
	54-1G07	NA	1.70E+04	No Diss
	54-4G07	9.74E-08	5.19E+04	5.06E-03
	54-4H03	4.09E-08	9.55E+04	3.90E-03
	ClonB UCA	1.83E-08	1.43E+05	2.61E-03
	58-6E11	4.59E-09	1.12E+05	5.16E-04
	58-6B09	7.80E-09	1.67E+05	1.30E-03
	58-6E04	3.50E-09	2.34E+05	8.19E-04
	58-6F03	NA	2.08E+05	No Diss
	54-1G05	NA	1.81E+05	No Diss
H1 NC99	ClonA UCA	No Binding		
	58-1C02	2.70E-08	8.30E+03	2.30E-04
	54-1B01	1.40E-07	5.40E+03	7.50E-04
	54-1G07	9.90E-09	5.90E+03	5.80E-05
	54-4G07	2.10E-06	1.20E+04	2.40E-02
	54-4H03	3.20E-06	1.70E+04	5.30E-02
	ClonB UCA	3.21E-08	4.97E+04	1.60E-03
	58-6E11	1.80E-09	6.70E+04	1.20E-04
	58-6B09	1.50E-08	5.40E+04	8.10E-04
	58-6E04	NA	8.70E+04	No Diss
	58-6F03	3.10E-11	8.60E+04	2.60E-06
	54-1G05	5.90E-10	6.50E+04	3.80E-05
H5 IN05	ClonA UCA	No Binding		
	58-1C02	1.59E-07	9.30E+03	1.48E-03
	54-1B01	2.76E-07	7.47E+03	2.06E-03
	54-1G07	2.21E-07	7.11E+03	1.57E-03
	54-4G07	No Binding		
	54-4H03	3.67E-06	3.09E+04	1.13E-01
	ClonB UCA	5.79E-07	2.09E+04	1.21E-02
	58-6E11	1.76E-07	3.50E+04	6.14E-03
	58-6B09	7.00E-08	4.09E+04	2.86E-03
	58-6E04	5.40E-09	1.51E+05	8.16E-04
	58-6F03	2.00E-09	1.04E+05	2.08E-04
	54-1G05	8.64E-09	8.37E+04	7.24E-04
H2 SI57	ClonA UCA	No Binding		
	58-1C02	NA	2.14E+04	No Diss
	54-1B01	4.47E-07	6.00E+03	2.68E-03
	54-1G07	NA	6.42E+03	No Diss
	54-4G07	6.66E-07	1.95E+04	1.30E-02
	54-4H03	8.04E-07	1.21E+04	9.71E-03
	ClonB UCA	1.45E-07	5.99E+04	8.70E-03
	58-6E11	9.52E-08	5.14E+04	4.89E-03
	58-6B09	1.22E-08	7.25E+04	8.80E-04
	58-6E04	2.15E-08	1.14E+05	2.45E-03
	58-6F03	2.21E-09	1.17E+05	2.58E-04
	54-1G05	1.77E-08	7.40E+04	1.31E-03
H9 HK99	ClonA UCA	No Binding		
	58-1C02	No Binding		
	54-1B01	No Binding		
	54-1G07	No Binding		
	54-4G07	No Binding		
	54-4H03	No Binding		
	ClonB UCA	No Binding		
	58-6E11	No Binding		
	58-6B09	No Binding		
	58-6E04	3.74E-07	9.50E+04	3.55E-02
	58-6F03	5.60E-07	2.27E+04	1.27E-02
	54-1G05	1.87E-07	1.29E+05	2.40E-02

Table S2, related to Figure 2. X-ray data collection and refinement statistics

**Data collection**	1G05 Apo	1G05 with H1 HA	4H03 Apo	4H03 with H1 HA
Beamline	APS 23ID-D	APS 23ID-D	APS 23ID-B	APS 23ID-B
Wavelength (Å)	1.0332	1.0332	1.0332	1.0332
Space group	$P22_12_1$	P321	$P22_12_1$	$C222_1$
Unit cell parameters (Å)	a=60.9, b=79.1, c=107.6	a=b=187.4, c=132.5	a=60.4, b=80.1, c=107.3	a=231.5, b=259.2, c=165.1
Resolution (Å)	50-1.65 (1.71-1.65)[a]	50-4.20 (4.60-4.20)[a]	50-1.80 (1.87-1.80)[a]	50-3.50 (3.61-3.50)[a]
Unique Reflections	62,699 (6,106)[a]	19,952 (4,703)[a]	48,821 (5,325)[a]	62,420 (5,623)[a]
Redundancy	12.8 (12.3)[a]	19.2 (19.1)[a]	7.6 (7.1)[a]	6.0 (6.3)[a]
Completeness (%)	99.3 (98.3)[a]	99.9 (100.0)[a]	100.0 (100.0)[a]	99.8 (99.8)[a]
$\langle I/\sigma_I \rangle$	27.5 (2.1)[a]	4.8 (2.6)[a]	34.0 (2.1)[a]	9.4 (1.4)[a]
R_{sym}[b]	0.08 (0.86)[a]	0.40 (0.99)[a]	0.06 (0.71)[a]	0.19 (1.14)[a]
R_{pim}[b]	0.02 (0.25)[a]	0.09 (0.23)[a]	0.02 (0.29)[a]	0.08 (0.48)[a]
$CC_{1/2}$[c]	1.00 (0.96)[a]	0.99 (0.97)[a]	1.00 (0.95)[a]	0.99 (0.90)[a]
Z_a[d]	1	1	1	3
**Refinement statistics**				
Resolution (Å)	50-1.65	50-4.20	50-1.80	50-3.50
Reflections (work)	59,570	18,836	46,393	59,210
Reflections (test)	3,082	923	2,359	3,149
R_{cryst}(%)[e] / R_{free}(%)[f]	15.8 / 18.8	32.3 / 38.9	16.3 / 20.4	17.9 / 23.2
No. of atoms				
Protein				
HA	-	3,921	-	11,759
Fab	3,524	3,507	3,577	10,194
Water	629	-	456	-
Glycan	-	14	-	448
Solvent[g]	70	-	12	-
Average *B*-value (Å^2)				
Protein				
HA	-	124	-	118
Fab	28	148	38	131
Water	43	-	50	-
Glycan	-	114	-	167
Solvent[g]	79	-	43	-
Wilson *B*-value (Å^2)	20	92	28	85
**RMSD from ideal geometry**				
Bond length (Å)	0.006	0.003	0.007	0.003
Bond angle (°)	0.84	0.61	1.00	0.55
**Ramachandran statistics (%)**[h]				
Favored	97.7	94.2	97.6	96.0
Outliers	0.0	0.0	0.2	0.1
**PDB code**	6WIY	6WIZ	6WJ0	6WJ1

[a] Numbers in parentheses refer to the highest resolution shell.

[b] $R_{sym} = \Sigma_{hkl} \Sigma_i | I_{hkl,i} - \langle I_{hkl} \rangle | / \Sigma_{hkl} \Sigma_i I_{hkl,i}$ and $R_{pim} = \Sigma_{hkl} (1/(n-1))^{1/2} \Sigma_i | I_{hkl,i} - \langle I_{hkl} \rangle | / \Sigma_{hkl} \Sigma_i I_{hkl,i}$, where $I_{hkl,i}$ is the scaled intensity of the ith measurement of reflection h, k, l, $\langle I_{hkl} \rangle$ is the average intensity for that reflection, and *n* is the redundancy.

[c] $CC_{1/2}$ = Pearson correlation coefficient between two random half datasets.

[d] Z_a is the number of Fab or Fab-HA protomers per crystallographic asymmetric unit.

[e] $R_{cryst} = \Sigma_{hkl} | F_o - F_c | / \Sigma_{hkl} | F_o | \times 100$, where F_o and F_c are the observed and calculated structure factors, respectively.

[f] R_{free} was calculated as for R_{cryst}, but on a test set comprising 5% of the data excluded from refinement.

[g] Solvent includes glycerol.

[h] Calculated with MolProbity (Chen et al., 2010).

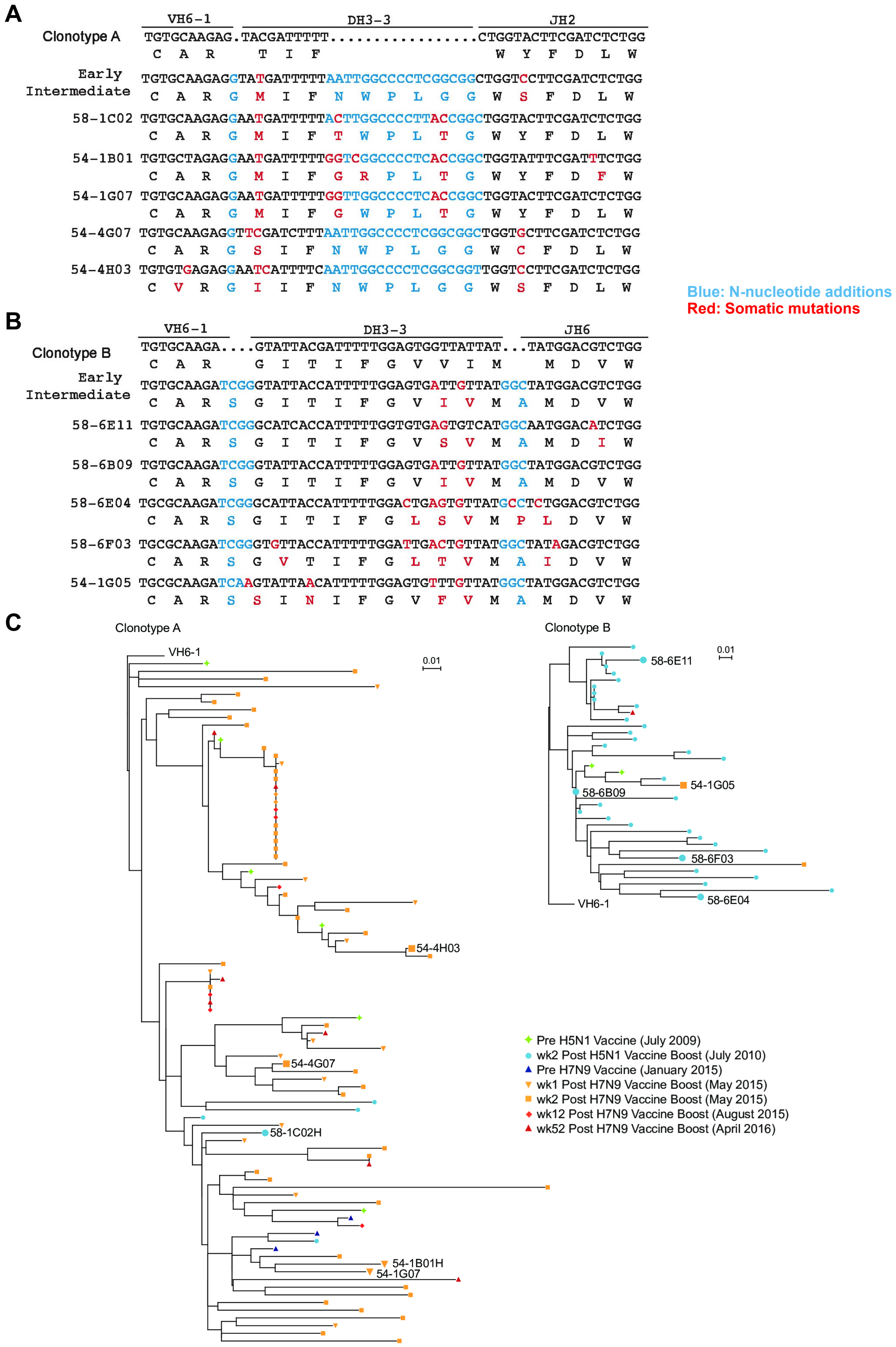

Figure S1, related to Figure 1. Phylogenetic analysis of Clonotypes A and B. (A-B) Sequence of the V-D-J junction of members in **(A)** clonotype A and **(B)** clonotype B, with putative gene segments indicated. N-nucleotide additions are colored blue, somatic mutations are colored red. **(C)** Phylogenetic analysis was performed on the immunoglobulin heavy chain sequence of Clonotype A and B members amplified from B cells detected at various time points before and after H5N1 and H7N9 vaccination as indicated in the legend.

		HA1															
		8	10	11	18	20	21	28	30	37	38	39	40	315	318	319	325
Group 1	A/California/04/2009 (H1N1)	A	A	D	H	N	N	T	L	T	H	S	V	R	T	G	S
	A/New Caledonia/20/1999 (H1N1)	T	A	D	H	N	N	T	L	T	H	S	V	R	T	G	S
	A/Canada/720/2005 (H2N2)	-	G	D	H	N	N	T	L	T	H	A	K	V	T	G	Q
	A/Indonesia/5/2005 (H5N1)	-	S	D	H	N	N	T	M	T	H	A	Q	V	T	G	Q
	A/Hong Kong/1073/1999 (H9N2)	S	A	D	Q	T	N	T	T	T	H	A	K	K	V	G	A
Group 2	A/Perth/16/2009 (H3N2)	N	T	A	H	V	P	T	T	T	N	A	T	K	T	G	E
	A/Texas/50/2012 (H3N2)	N	T	A	H	V	P	T	T	T	N	A	T	K	T	G	E
	A/Shanghai/02/2013 (H7N9)	T	A	D	H	V	S	T	T	V	N	A	T	L	T	G	E
Epitope	54-4H03				■	■	■				■	■	■		■		
	54-1G05				■	■	■			■	■		■		■	■	
	56.a.09				■						■				■		■
	MEDI8852	■	■	■				■	■					■			

		HA2																																
		15	16	18	19	20	21	24	25	26	30	31	32	33	34	35	36	37	38	39	41	42	43	45	46	48	49	52	53	56	146	150	153	154
Group 1	A/California/04/2009 (H1N1)	T	G	V	D	G	W	Y	H	H	Q	G	S	G	Y	A	A	D	L	K	T	Q	N	I	D	I	T	V	N	I	N	E	K	N
	A/New Caledonia/20/1999 (H1N1)	T	G	V	D	G	W	Y	H	H	Q	G	S	G	Y	A	A	D	Q	K	T	Q	N	I	N	I	T	V	N	I	N	E	K	N
	A/Canada/720/2005 (H2N2)	Q	G	V	D	G	W	Y	H	H	Q	G	S	G	Y	A	A	D	K	E	T	Q	K	F	D	I	T	V	N	I	D	N	K	N
	A/Indonesia/5/2005 (H5N1)	Q	G	V	D	G	W	Y	H	H	Q	G	S	G	Y	A	A	D	K	E	T	Q	K	I	D	V	T	V	N	I	N	E	R	N
	A/Hong Kong/1073/1999 (H9N2)	P	G	V	A	G	W	F	Q	H	Q	G	V	G	M	A	A	D	R	D	T	Q	K	I	D	I	T	V	N	V	D	E	R	N
Group 2	A/Perth/16/2009 (H3N2)	E	G	V	D	G	W	F	R	H	E	G	R	G	Q	A	A	D	L	K	T	Q	A	I	D	I	N	L	N	I	N	G	R	N
	A/Texas/50/2012 (H3N2)	E	G	V	D	G	W	F	R	H	E	G	R	G	Q	A	A	D	L	K	T	Q	A	I	D	I	N	L	N	I	N	G	R	N
	A/Shanghai/02/2013 (H7N9)	E	G	I	D	G	W	F	R	H	Q	G	E	G	T	A	A	D	Y	K	T	Q	S	I	D	I	T	L	N	I	D	A	R	N
Epitope	54-4H03	■	■	■	■	■	■								■	■	■		■		■	■		■	■	■	■	■	■	■	■	■	■	
	54-1G05	■	■	■	■	■	■	■						■	■	■	■	■	■	■	■	■	■	■	■	■	■	■				■	■	
	56.a.09	■	■	■	■	■	■		■		■				■	■	■		■	■	■	■		■	■	■	■				■	■	■	■
	MEDI8852	■	■	■	■	■	■			■	■	■	■	■	■				■	■	■	■	■	■	■	■	■	■			■			

Figure S2, related to Figure 2. Epitopes of V_H6-1-encoded HA stem-directed antibodies. Amino-acid variants in different strains are shown for the epitope residues of 54-4H03, 54-1G05, 56.a.09 (Joyce et al., 2016), and MEDI8852 (Kallewaard et al., 2016). The epitope residues for each antibody are indicated by the black squares at the bottom. HA1 residue 38 is glycosylated in group 2 HAs.

A

```
                                       CDR L1
MEDI8852: DIQMTQSPSSLSASVGDRVTITCRTSQSL-
56.a.09:  EIVLTQSPGTLSLSPGERATLSCRASQSVA
1G05:     EIVLTQSPGTLSLSPGERVTLSCRASQTVY
4H03:     EIVLTQSPGTLSLSPGDSATLSCRASQSVA

                                CDR L2
MEDI8852: SSYTHWYQQKPGKAPKLLIYAASSRGSGVP
56.a.09:  SSYLAWYQQKPGQAPRLLIYGASSRATGVP
1G05:     NSYLAWYQQKPGQAPTLLIYGTSTRATGVP
4H03:     SSYLAWYQQKPGQSPRLLIYATINRAADIP
            ↑
            32

MEDI8852: SRFSGSGSGTDFTLTISSLQPEDFATYYCQ
56.a.09:  DRFSGSGSGTDFILTISRLEPEDFAVYYCQ
1G05:     DRFSGSGSGTVFTLTISRLEPEDFAVYFCQ
4H03:     DRFSGSGSGTDFALTISRLEPEDFAVYYCQ

            CDR L3
MEDI8852: Q------SRTFGQGTKVEIK
56.a.09:  QYDG--SQYTFGQGTKLEIK
1G05:     QYSTSPRALTFGGGTKVEIK
4H03:     QFDSS-SMYTFGQGTKLEIT
```

B

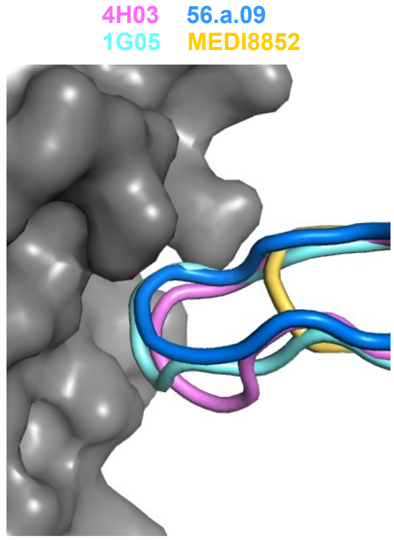

Figure S3, related to Figure 3. Comparison of the conformations of CDR L3 among different V_H6-1-encoded bnAbs. (A) Alignment of the light chain sequences from 54-4H03, 54-1G05, 56.a.09 (Joyce et al., 2016), and MEDI8852 (Kallewaard et al., 2016). The regions of the sequence that correspond to CDR L1, L2, and L3 (Kabat numbering scheme) are indicated. Paratope residues are highlighted in red. **(B)** The lengths of the CDR L3 in 54-4H03, 54-1G05, and 56.a.09 are similar, but are much longer than that of MEDI8852.

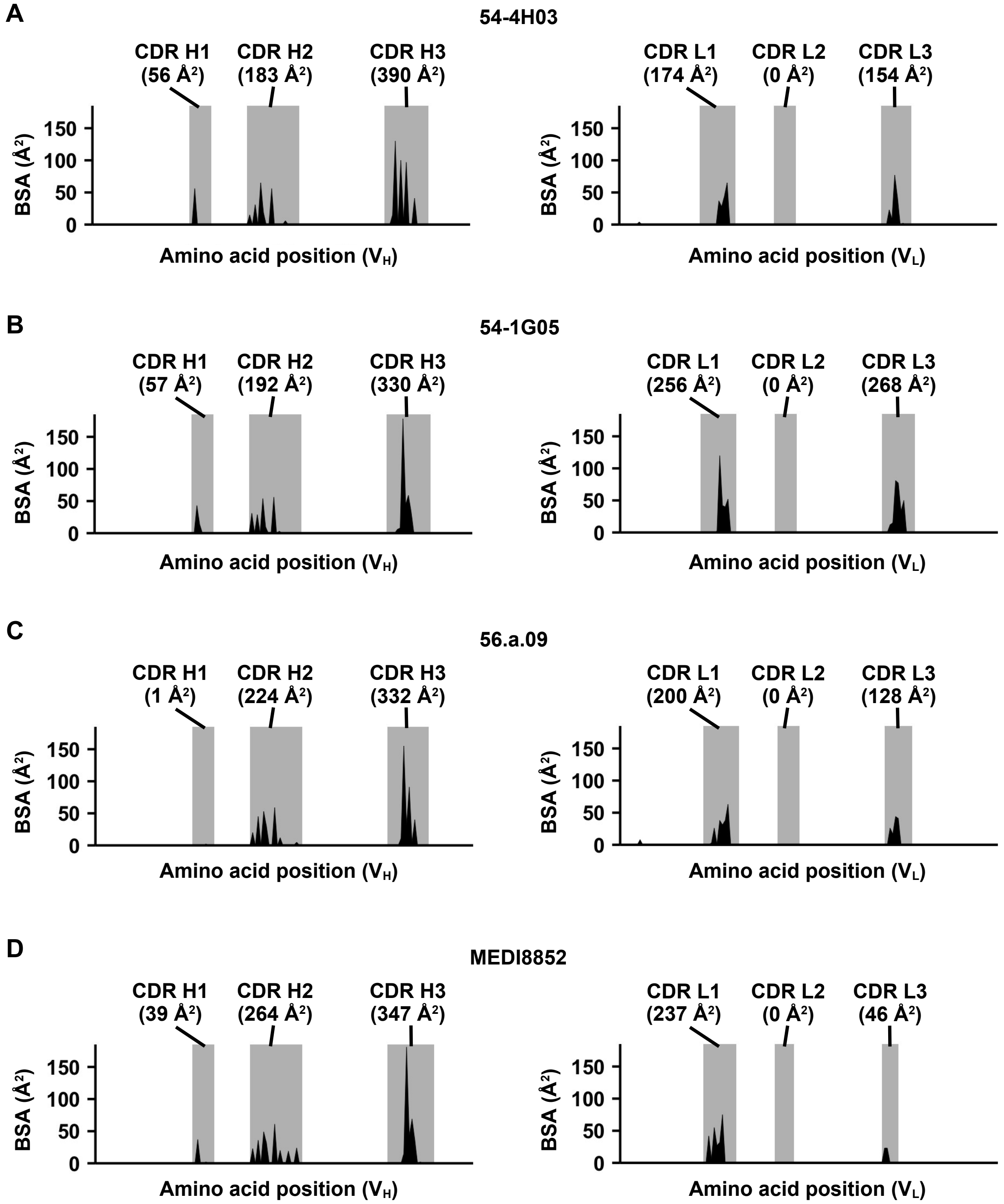

Figure S4, related to Figure 3. Buried surface area (BSA) of paratopes of V_H6-1-encoded bnAbs upon binding to HA. Buried surface area (BSA) upon binding for each residue in **(A)** 54-4H03 Fab, **(B)** 54-1G05 Fab, **(C)** 56.a.09 Fab, and **(D)** MEDI8852 Fab, is plotted along the residue position in the sequence. The regions corresponding to the CDRs (based on Kabat numbering) are shaded in grey. The BSA of each CDR is indicated in parentheses.

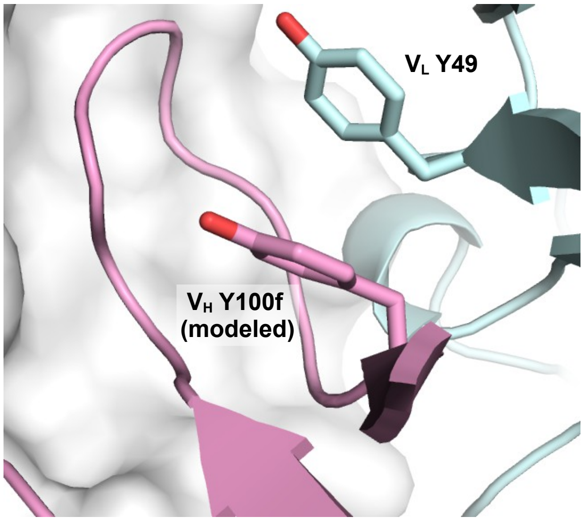

Figure S5, related to Figure 6. Potential π–π stacking interaction between V_H Y100f and V_L Y49 in clonotype A. Somatic mutation V_H S100fY is modeled on 54-1G05, and can form a π–π stacking interaction with Tyr49 of the light chain .

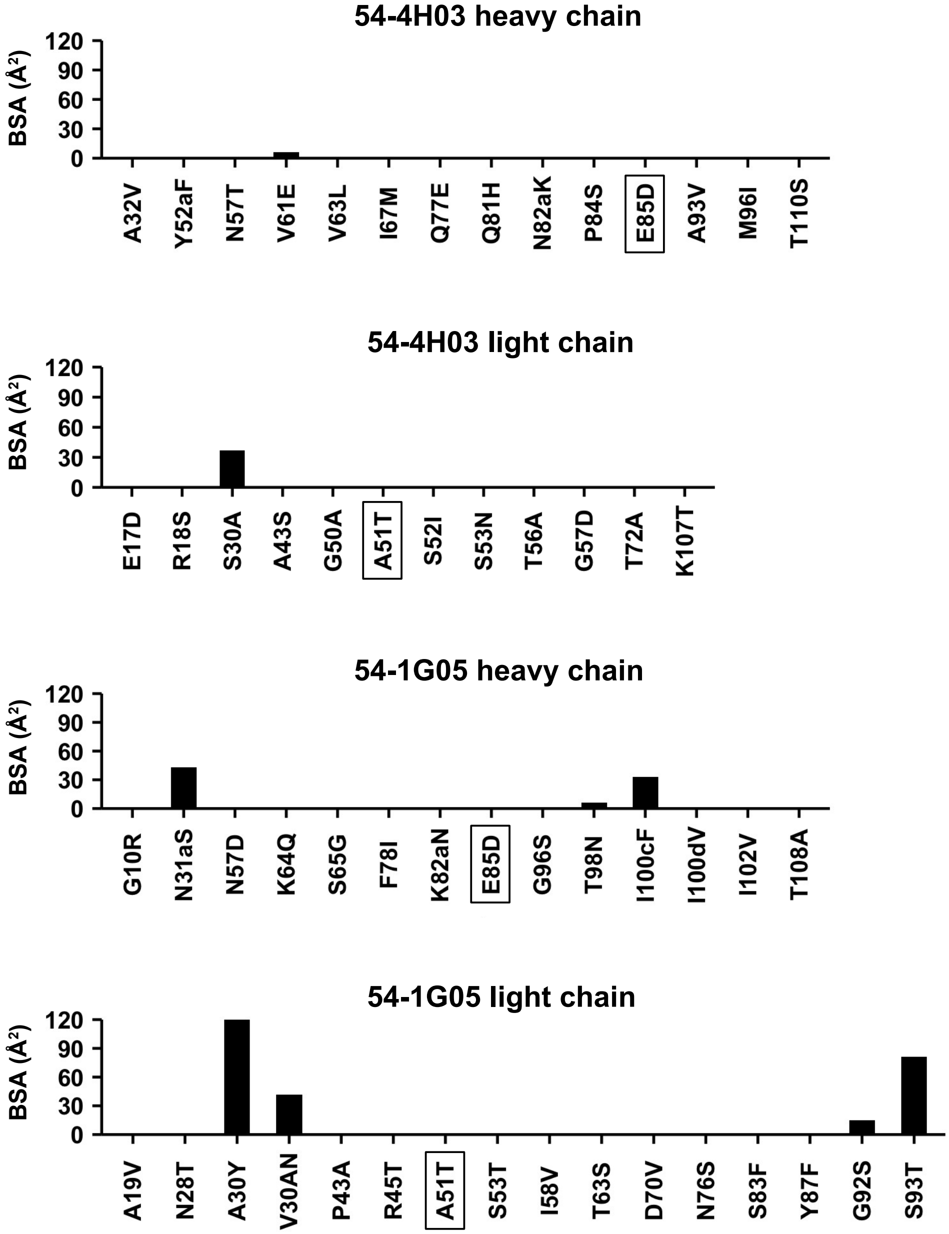

Figure S6, related to Figure 6 and Figure 7. Buried surface area of somatic mutations. The buried surface area (BSA) of each somatic mutation in 54-4H03 and 54-1G05 is shown. BSA is calculated as (solvent accessible surface area of the apo form) – (solvent accessible surface area of the bound form). Somatic mutations that are common between 54-4H03 and 54-1G05 are boxed.

A

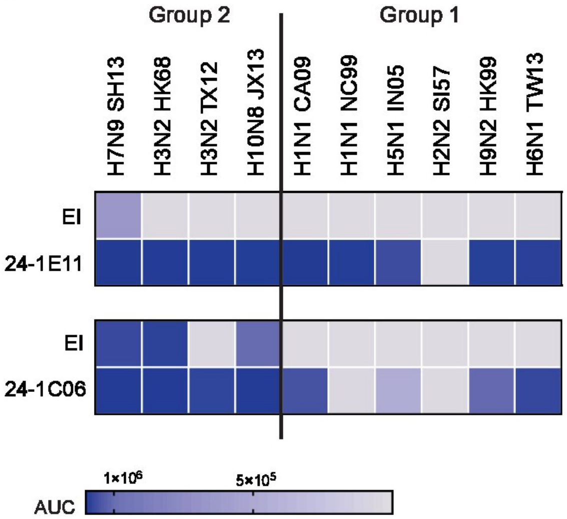

B

CDR H3 for 24-1E11: CARSGLLYFGMLLHFDYW
CDR H3 for 24-1C06: CARAQSLTIFGVVINLDSW

Figure S7, related to Figure 1. Additional analysis of V_H6-1-encoded bnAbs. **(A)** V_H6-1/V_κ3-20-encoded antibodies 24-1E11 and 24-1C06 were both isolated from another donor. The binding of these two antibodies as well as their corresponding early intermediate (EI) to recombinant HA from different subtypes were measured by MSD. The area under the binding curve (AUC) of each mAb for each HA is indicated by color as shown in the legend. **(B)** The amino-acid sequences for CDR H3 of 24-1E11 and 24-1C06.
